# Anatomy, Flow Cytometry, and X-Ray Tomography Reveal Tissue Organization and Ploidy Distribution in Long-Term *In Vitro* Cultures of *Melocactus* Species

**DOI:** 10.3389/fpls.2020.01314

**Published:** 2020-08-31

**Authors:** Gabriela Torres-Silva, Elyabe Monteiro Matos, Ludmila Freitas Correia, Evandro Alexandre Fortini, Wellington Santos Soares, Diego Silva Batista, Caio Gomide Otoni, Aristéa Alves Azevedo, Lyderson Facio Viccini, Andréa Dias Koehler, Sheila Vitória Resende, Chelsea Dvorak Specht, Wagner Campos Otoni

**Affiliations:** ^1^Laboratory of Plant Tissue Culture II—BIOAGRO, Plant Biology Department, Federal University of Viçosa (UFV), Viçosa, Brazil; ^2^Laboratory of Genetics and Biotechnology, Department of Biology, Federal University of Juiz de Fora (UFJF), Juiz de Fora, Brazil; ^3^Department of Agriculture, Federal University of Paraíba (UFPB), Bananeiras, Brazil; ^4^Institute of Chemistry, University of Campinas (UNICAMP), Campinas, Brazil; ^5^Laboratory of Plant Anatomy, Viçosa, Brazil; ^6^Institute of Biology, Federal University of Bahia (UFBA), Salvador, Brazil; ^7^Plant Biology Section, School of Integrative Plant Science, Cornell University, Ithaca, NY, United States

**Keywords:** cacti, endangered species, endocycle, endoreduplication, genome size, mixoploidy

## Abstract

Cacti have a highly specialized stem that enables survival during extended dry periods. Despite the ornamental value of cacti and the fact that stems represent the main source of explants in tissue culture, there are no studies on their morpho-anatomical and cytological characteristics in *Melocactus*. The present study seeks to address the occurrence of cells with mixed ploidy level in cacti tissues. Specifically, we aim to understand how *Melocactus* stem tissue is organized, how mixoploidy is distributed when present, and whether detected patterns of ploidy change after long periods of *in vitro* culture. To analyze tissue organization, *Melocactus glaucescens* and *Melocactus paucispinus* plants that had been germinated and cultivated *in vitro* were analyzed for stem structure using toluidine blue, Xylidine Ponceau, Periodic Acid Schiff, ruthenium red, and acid floroglucin. To investigate patterns of ploidy, apical, medial, and basal zones of the stem, as well as, periphery, cortex, and stele (vascular tissue and pith) regions of the stem and root apexes from four- and ten-year old cultured *in vitro* were analyzed by flow cytometry. X-ray micro-computed tomography (XRµCT) was performed with fragments of stems from both species. The scarcity of support elements (*i.e.*, sclereids and fibers) indicates that epidermis, hypodermis, and wide-band tracheids present in cortical vascular bundles and stele, as well as water stored in aquifer parenchyma cells along the cortex, provide mechanical support to the stem. Parenchyma cells increase in volume with a four-fold increase in ploidy. *M. glaucescens* and *M. paucispinus* exhibit the same pattern of cell ploidy irrespective of topophysical region or age, but there is a marked difference in ploidy between the stem periphery (epidermis and hypodermis), cortex, stele, and roots. Mixoploidy in *Melocactus* is not related to the age of the culture, but is a developmental trait, whereby endocycles promote cell differentiation to accumulate valuable water.

## Key Message

In this study, we provide new insights on variations in endoreduplication-derived ploidy levels associated with topophysical zones in stems of two endangered *Melocactus* species; this evidence will improve our understanding on how cacti survive in xeric environments.

## Highlights

- *Melocactus glaucescens* and *M. paucispinus* are diploid but stems show mixoploidy.
- Mixoploidy of the two cacti is not related to the age of *in vitro* cultures.
- Mixoploidy is a developmental characteristic that allows for tissue differentiation.
- The small *Melocactus* genome in the roots enables rapid cell division cycles.
- Endoreduplication in cortex cells enables expansion to optimize water storage.

## Introduction

Cacti are highly adapted to xeric environments, owing to physiological modifications such as the Crassulacean Acid Metabolism (CAM) or morphological adaptations (Anderson, 2001). The latter affect particularly the stem, which consists mostly of water-storage aquifer parenchyma tissue and usually has a thick cuticle that limits water loss and a hypodermis (Anderson, 2001; Arruda et al., 2004; Ventura-Aguilar et al., 2017).

The specialized stems play an important role in their taxonomy as their colors and shapes often provide the characteristics that distinguish subfamilies and genera within Cactaceae (Anderson, 2001). Moreover, the stem is used extensively in vegetative propagation, by either removing branches or using sectioned parts as explants in tissue culture (Lema-Rumińska and Kulus, 2014; Pérez-Molphe-Balch et al., 2015).

Some species of Cactaceae such as species of the genus *Melocactus* do not branch (Machado, 2009). This limits opportunities for vegetative propagation, making them particularly susceptible to overharvesting during the removal of whole specimens from their natural populations for commercialization as ornamental plants (Goettsch et al., 2015; Pérez-Molphe-Balch et al., 2015). This has led to the development of conservation strategies that rely on *in vitro* propagation of *Melocactus* species (Resende et al., 2010; Torres-Silva et al., 2018; Santos et al., 2020). Tissue culture techniques represent an attractive form of propagation for this genus, even though their slow growth means that some species require about ten years to reach reproductive age (Machado, 2009; Lema-Rumińska and Kulus, 2014; Pérez-Molphe-Balch et al., 2015).

During *in vitro* propagation of *Melocactus glaucescens*, the apical segment is removed and the stem is sectioned transversally into discs (Torres-Silva et al., 2018). Subsequently, the shoots arise from the areola region of *M. glaucescens* stems grown in culture medium with or without plant growth regulators (PGRs) (Torres-Silva et al., 2018). This is the main form of cacti propagation, called areola activation (Lema-Rumińska and Kulus, 2014; Pérez-Molphe-Balch et al., 2015).

In spite of the growing interest for cacti as ornamental plants and the fact that the main form of propagation is *via* the stem, very few studies have investigated anatomical and cytological organization of these plants and explants. A recent cytological characterization of *Copiapoa tenuissima* (Cactaceae) performed by Lema-Rumińska (2011) revealed the presence of cells with different ploidy levels and demonstrated that ploidy increased with tissue specialization. This result was attributed to age and the use of PGRs in the culture medium. A study of *in vitro* shoot production of *M. glaucescens* revealed the occurrence of morphological and genetic variation in shoots derived from culture media with or without PGRs (Torres-Silva et al., 2018). However, the lack of correlation between the morphological and genetic variation among shoots demonstrates the need to better understand the cytological organization of *Melocactus* stems.

The ability to maintain cells with various DNA contents in somatic tissues is called mixoploidy and is common in tissues of succulents and cacti (Lee et al., 2009; Lema-Rumińska, 2011; Scholes and Paige, 2015). DNA content of 2C, 4C, 8C, and 16C has been reported in various Cactaceae, such as *Consolea tenuissima*, *C. corallicola*, *C. picardea*, *C. moniliformis*, *Mammillaria san-angelis*, *M. albilanata*, *M. crucigera*, *M. dixanthocentron*, *M. flavicentra*, *M. haagena*, *M. huitzilopochtl*, *M. supertexta*, *Opuntia heliabravoana*, *O. joconostle*, *O. matudae*, *O. oligacantha*, *O. hyptiacantha*, and *O. tomentosa* (Palomino et al., 1999; Del Angel et al., 2006; Negrón-Ortiz, 2007; Lema-Rumińska, 2011; Palomino et al., 2016). However, only a few studies so far have investigated how mixoploidy is distributed among cacti tissues (Lema-Rumińska, 2011).

Endocycle-driven endopolyploidization results in cells with mixed ploidy due to repeated endocycles, whereby an increase in genomic content occurs without concomitant cytokinesis. The implications of endoreduplication for physiology, growth, and development in plants have been comprehensively reviewed elsewhere (Traas et al., 1998; Joubès and Chevalier, 2000; Lee et al., 2009; Breuer et al., 2010; Breuer et al., 2014; Edgar et al., 2014; Scholes and Paige, 2015; Ochatt and Abirached-Darmency, 2020). The biological function of endocycles is related to the formation of specialized cell types, such as trichomes and vascular elements; cells with higher metabolic activity such as embryo-associated cells; storage tissues, such as cotyledonary and aquifer parenchyma cells, which occur through different plant organs; and on auxin signaling and hence on morphogenetic competence (De Rocher et al., 1990; Palomino et al., 1999; Lee et al., 2009; Breuer et al., 2010; Breuer et al., 2014; Bourge et al., 2018; Ochatt and Abirached-Darmency, 2020).

This work aims to collect cytological, histochemical, and anatomical information to determine whether *Melocactus* stems are mixoploid, how this mixoploidy is distributed through the stem tissues, and how the pattern of ploidy may be altered by long periods of *in vitro* culture. Histological, flow cytometric, and X-ray micro-computed tomography (XRµCT) approaches were employed. The study provides a better understanding of the morphological and cytogenetic alterations that accompany *in vitro* propagation of *Melocactus* species.

## Materials and Methods

### Plant Material

Seeds were collected in Morro do Chapéu from natural populations of *M. glaucescens* (11°29′38.4′′ S; 41°20′22.5′′ W) and *Melocactus paucispinus* (11°33′52.0′′ S; 41°10′38.8′′ W) in February of 2008 and 2014. These species are endemic to Bahia state, eastern Brazil and are listed as endangered by the Convention on International Trade in Endangered Species of Wild Fauna and Flora (UNEP-WCMC (Comps.), 2014).

To establish *in vitro* cultures, the seeds were surface-sterilized by immersion in 96% ethanol for 1 min, 2% commercial bleach (2.5% active chlorine; SuperGlobo^®^, Contagem, MG, Brazil) for 10 min, and three washes in sterile water under aseptic conditions. Afterwards, seeds were germinated in 350-ml capacity flasks (Model AZ200; Embalagens Rio, Rio de Janeiro, RJ, Brazil) containing 50 ml of MS culture medium (Murashige and Skoog, 1962), at quarter-strength salt. The flasks were covered with 67.5 mm diameter transparent polypropylene lids (TC-003-2012, Ralm^®^, São Bernardo do Campo, SP, Brazil).

After 21 days, seedlings were recultured on a six months-basis in 585-ml capacity flasks (Model AZF 350 flasks; Embalagens Rio, Rio de Janeiro, Brazil) containing 100 ml MS culture medium at half-strength salt concentration. The flasks were capped with transparent polypropylene lids. All culture media were devoid of plant growth regulators but supplemented with 15 g L^−1^ sucrose and solidified with 7 g L^−1^ agar (A296 Plant TC, PhytoTechnology Lab^®^, Shawnee Mission, KS, USA), with pH adjusted to 5.7 before autoclaving at 121°C and 1.5 atm for 20 min.

Cultures were maintained at 25 ± 2°C under two fluorescent lamps (110 W, HO Sylvania T12, Wilmington, MA, USA) with photosynthetically active radiation levels of 60 µmol m^−2^ s^−1^ and a 16/8 h light/dark photoperiod. All samples were selected from the germplasm collection of the Laboratory of Plant Tissue Culture II (BIOAGRO) of the Federal University of Viçosa (Brazil).

### Anatomy

For anatomical characterization, samples from the medial zone of the stem were collected from five plants of *M. glaucescens* and five plants of *M. paucispinus* that had been grown *in vitro* for four years. The samples were fixed in 0.1 M Karnovsky solution (Karnovsky, 1965) under −250 mm Hg vacuum for 1 h. The material was stored in this solution until handling, at which point it was dehydrated in an ethanol series and put again under −250 mm Hg vacuum. The samples were then embedded in methacrylate resin (Historesin^®^; Leica Biosystems Nussloch GmBH, Nussloch, Germany), after which transversal and longitudinal sections (average 5 µm in thickness) were made with a rotary microtome (RM 2155; Leica Biosystems Division, Buffalo Grove, IL, United States). The sections were placed on slides and stained with 0.05 vol% toluidine blue (Sigma-Aldrich Co, St, Louis, MO, United States) pH 4.4 for 10 min (O’Brien and McCully, 1981).

The slides were stained with Xylidine Ponceau (Sigma-Aldrich) solution to evidence proteins, Periodic Acid Schiff (PAS) for the detection of polysaccharides and ruthenium red to visualize pectin (Johansen, 1940). Slides of fresh material were submitted to acid floroglucin (Sigma-Aldrich) test to confirm the presence of lignin (Johansen, 1940). The material was observed under an optical microscope (AX70TRF; Olympus Optical). Images were taken with a digital camera (Spot Insightcolour 3.2.0, Diagnostic Instruments Inc.) using Spot Basic Image software.

### Flow Cytometry

**Figure 1:**
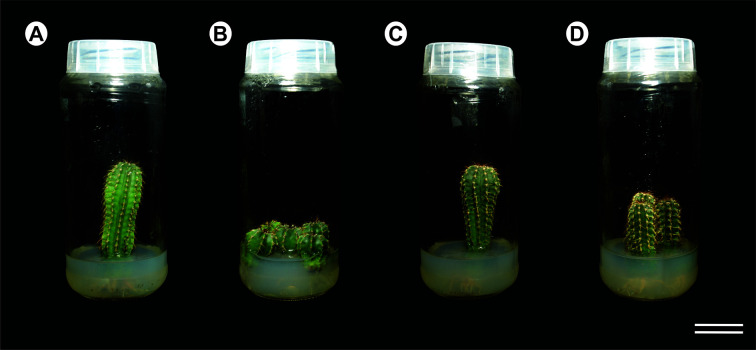
Detail of the long-term *in vitro* cultures of *Melocactus* species: **(A)** Ten- and four-year **(B)** old *in vitro* cultures of *Melocactus glaucescens*; **(C)** Ten- and four- years **(D)** old *in vitro* cultures of *M. paucispinus*.

**Figure 2:**
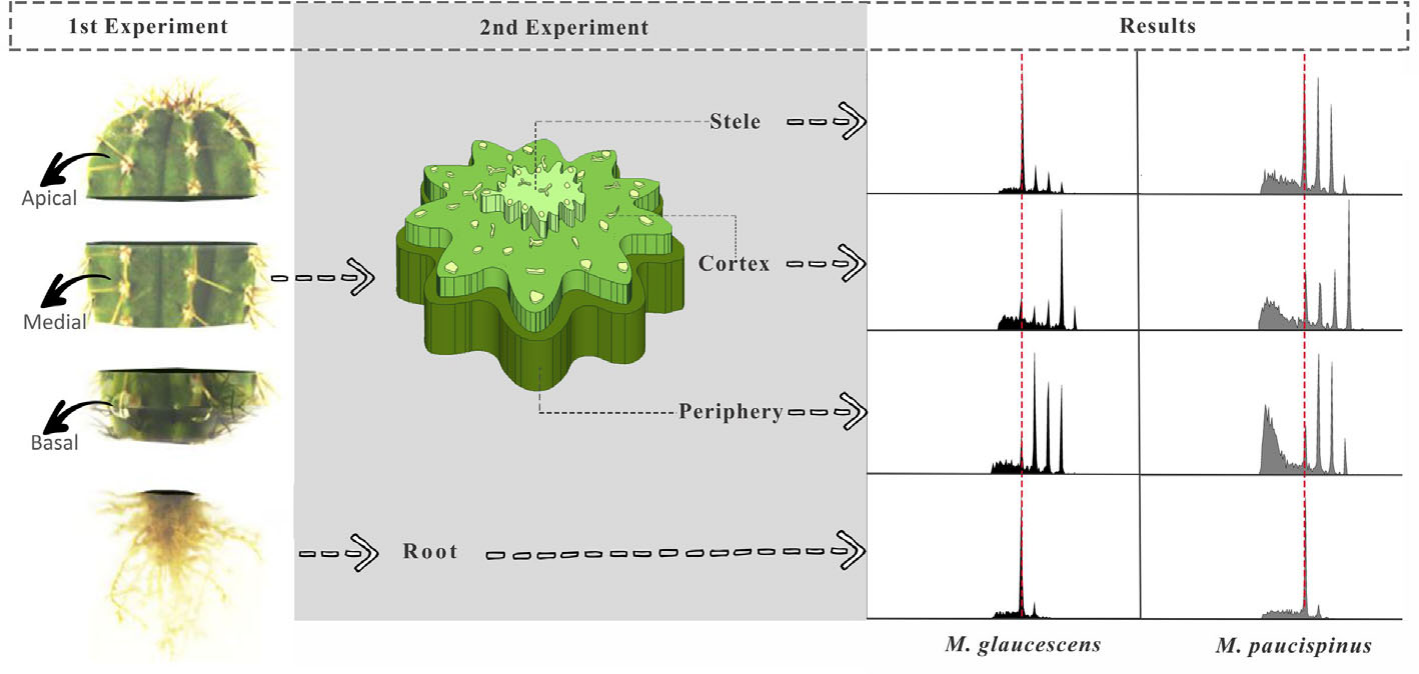
Diagram showing *Melocactus* stem segmentation for flow cytometry experiments and corresponding results. In the first experiment, the stems were sectioned transversally producing three longitudinal topophysical zones (apical, medial, and basal). In the second experiment, tissues of the stem were manually separated into stele, cortex region, and the outermost region of the stem (periphery), as well as the roots, and sorted according to age of *in vitro* cultures (ten and four years).

Two experiments were designed to investigate how ploidy varied through the *Melocactus* stems kept under *in vitro* culture in a MS-based medium PGR-free for 4 or 10 years (**Figure 1**). In the first experiment, the stems were sectioned transversally to generate three topophysical zones, namely apical, medial, and basal (**Figure 2**). Three plants of *M. glaucescens* and three of *M. paucispinus*, previously cultured *in vitro* for 10 years, were analyzed (**Figure 1**).

The second experiment analyzed the medial zone of the stem and the root apexes of cacti originating from *in vitro* cultures of two different ages (four and ten years) (**Figure 1**). The medial zone of the stem was separated manually in three samples with naked eyes. First a thin peripheral layer (approx. 1–2 mm) just below the thick layer of polymerized fatty acids (epidermis and hypodermis) was removed. Secondly a triangle with 1 cm of side was removed from the cortex section. Finally, the stele was recognized (naked eye) for its lighter color and removed manually from the center of the stem disc. A fourth region was included, the root (**Figure 1**).

For flow cytometry, approximately 20–30 mg of fresh tissue was chopped with a disposable steel razor blade in 1 ml WPB buffer to release the nuclei (Loureiro et al., 2007). *Solanum lycopersicum* ‘Stupické’ (2C DNA content = 1.96 pg) was used as an internal reference standard (Doležel et al., 1998). Previously macerated tissues were filtered through a 50 µM nylon mesh and collected in a polystyrene tube. The filtrate was stained with 25 µl propidium iodide solution (1 mg ml^−1^; Sigma-Aldrich). Samples were incubated at 4°C in the dark and examined after 30 min. At least 10,000 nuclei were analyzed in each sample thrice.

Data were acquired on a CytoFLEX instrument (Beckman Coulter Life Sciences, Indianopolis, IN, United States) at the Institute of Biological Sciences (ICB) of the Federal University of Juiz de Fora (UFJF) and then plotted and analyzed using CytExpert 2.0.1 software. Nuclear DNA content (pg) was estimated by the following equation (Doležel and Bartoš, 2005):

$$\begin{array}{l} \text{DNA}\;\left(2C\;\text{DNA}\right)\\ =\frac{G1\;\text{peak}\;\text{channel}\;\text{of}\;\text{sample}}{G1\;\text{peak}\;\text{channel}\;\text{of}\;S.\;lycopersicum\;\times \;1.96\;pg\;\left(S.\;lycopersicum\;\text{DNA}\;\text{content}\right)} \end{array}$$

### X-Ray Micro-Computed Tomography

The effect of increased ploidy on cell size in the different stem regions was assessed through the XRµCT of lyophilized *M. glaucescens* and *M. paucispinus* samples. The medial zone of the stems were frozen in liquid nitrogen and further lyophilized using an AdVantage 2.0 benchtop freeze dryer (VirTis, SP Scientific, Stone Ridge, NY) for 48 h.

The images were acquired on a SKYSCAN 1272 (Bruker microCT) scanner operating at a source voltage of 20 kV and a current of 175 µA. The NRecon software, version 1.6.10.4 (Micro Photonics, Inc) was used to reconstruct three-dimensional (3D) images from single-plane projections. The CTVox software, version 3.3 (Bruker microCT) was then applied for 3D viewing and sectioning.

Two-dimensional (2D) images were processed using the DataViewer software, version 1.5.6.2 (Bruker microCT). At least 200 undamaged cells were selected from the periphery and cortex of the stems, comprising volumes of interest from at least four cross-sectional layers throughout the sample’s height. The cells’ equatorial diameter (in case of isodiametric cells) or maximum Feret diameter (for irregular-shaped cells) was determined using the ImageJ software, version 1.52a (NIH).

### Statistical Analyses

Once data distribution was verified normal by the Lilliefors test (Conover, 1999), analysis of variance (ANOVA) was carried out in R statistical software to detect differences between topophysical zones (apical, medial, and basal) and regions (periphery, cortex, stele, and root apexes) of the stem, as well as between ages. Data describing the different regions and ages of the stem were arranged in a factorial scheme (four regions × two ages). The means were compared with Tukey’s test at 5% probability. Data for cell size were submitted to two-sample *t*-test at a confidence level of 95% in MINITAB^®^ Release 14 statistical software, with the null hypothesis being that the difference among the diameters of periphery and cortex cells within each species equals zero, *i.e.*, the populations are equal.

## Results

### Anatomy

**Figure 3:**
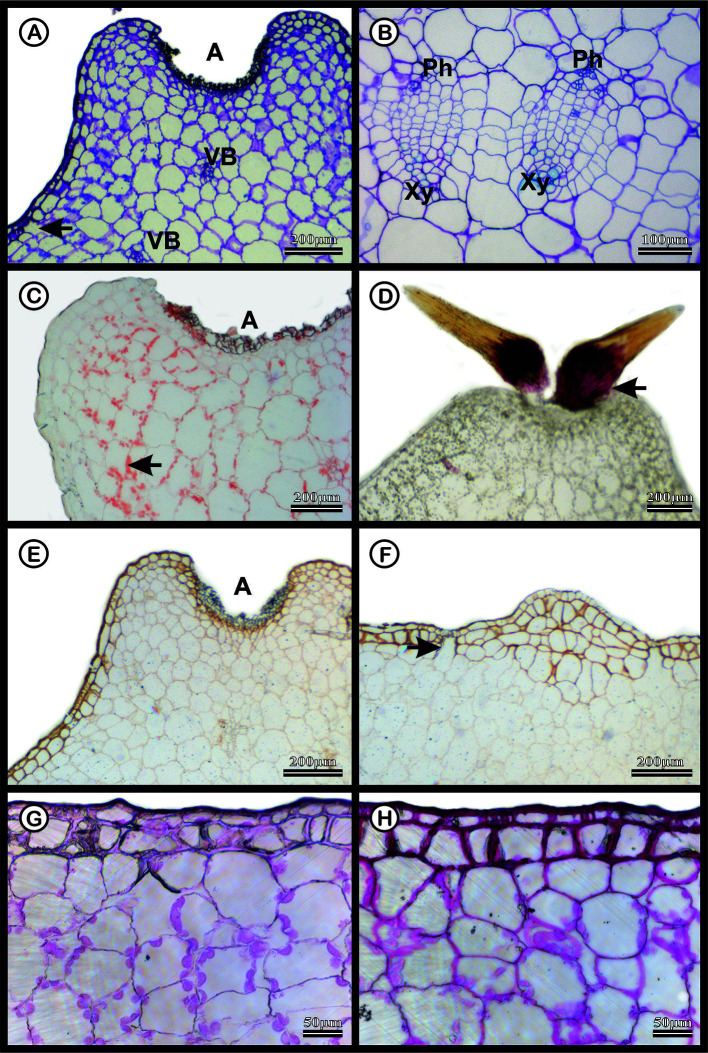
Anatomical characterization of *Melocactus glaucescens* medial zone of the stem of four-year old plants cultivated *in vitro*. **(A)** Toluidine blue staining of the areola region (A) showing cortical vascular bundles (VB). **(B)** Vascular system showing phloem (Ph) and xylem (Xy). **(C)** Xylidine Ponceau staining of the areola region (A); the arrow shows protein from nucleus. **(D)** Acid floroglucin staining showing the presence of lignin in the spine (arrow). **(E)** Ruthenium red staining of the areola region (A) and **(F)** uniseriate epidermis with substomatal cavities (arrow). **(G)** Negative and **(H)** positive PAS staining epidermis.

**Figure 4:**
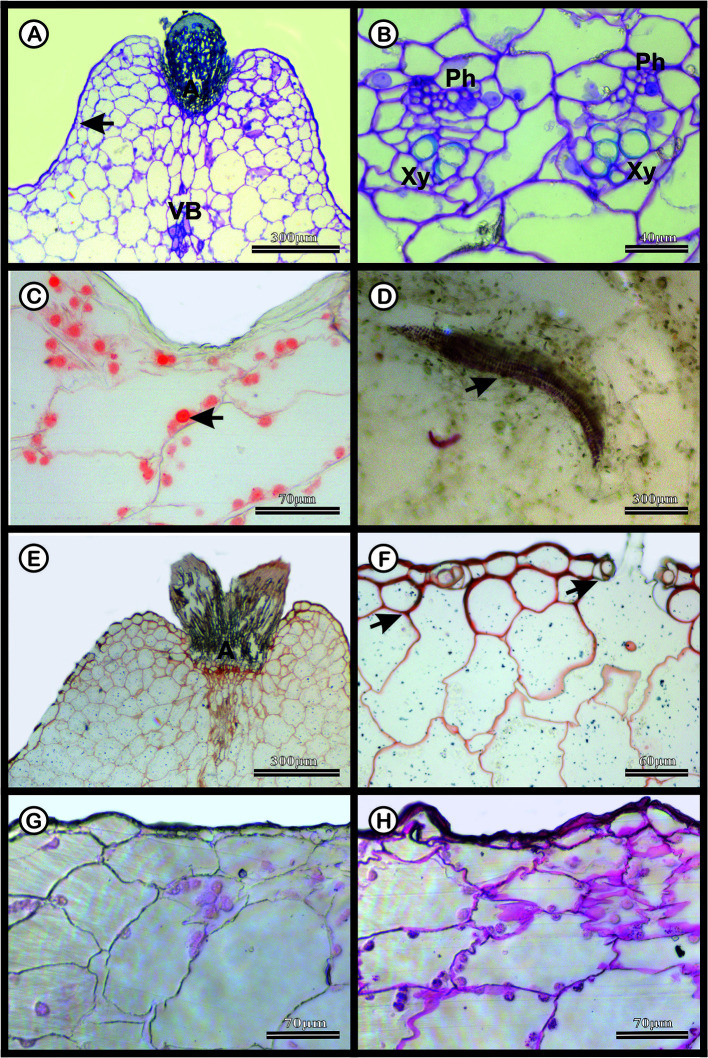
Anatomical characterization of *Melocactus paucispinus* medial zone of the stem of four-year old plants cultivated *in vitro*. **(A)** Toluidine blue staining of the areola region (A) showing cortical vascular bundles (VB). **(B)** Vascular system showing phloem (Ph) and xylem (Xy). **(C)** Xylidine Ponceau staining of the areola region; the arrow indicates the presence of proteins. **(D)** Acid floroglucin staining showing the presence of lignin in the secondary wall of the wide-band tracheid (arrow). **(E)** Ruthenium red staining of the areola region (A) and **(F)** uniseriate epidermis with stomatal cavities (arrows). **(G)** Negative and **(H)** positive PAS staining of the epidermis.

The dermal system of *M. glaucescens* and *M. paucispinus* consists of a single-layered with tabular cells (**Figures 3A** and **4A**). Stomata are located in the same plane as other epidermal cells, with substomatal cavities of varied depths (**Figures 3A, F** and **4A, F**). Beneath the epidermal layer is the collenchymatous hypodermis, which consists of cells with irregular wall thickening. This layer is more clearly distinguishable in *M. glaucescens* (**Figure 3A**) than in *M. paucispinus* (**Figure 4A**). The cortical region is composed of aquifer and chlorophyll parenchyma intercalated by cortical vascular bundles (**Figures 3A** and **4A**).

The vascular bundles are present in the cortex (**Figures 3A** and **4A**) and in the vascular cylinder (**Figures 3B** and **4B**), and are collateral in both species. The primary phloem contains a sieve tube element and parenchyma (**Figures 3B** and **4B**). The xylem consists of tracheids and parenchyma cells with few vessel elements.

In both species, Xylidine Ponceau stains positive to the proteins present in the nucleus (**Figures 3C** and **4C**). The acid floroglucin test (indicating lignin) was positive only in the spine region (**Figure 3D**) and in the secondary walls with helical thickening of the tracheids (**Figure 4D**). The tracheids are characterized by a secondary wall with helical thickening, known in the literature as wide-band tracheids (WBT) (**Figure 4D**). Ruthenium red and PAS staining revealed high amounts of pectin and polysaccharides respectively, in the epidermis and hypodermis, respectively, which were more accentuated in *M. glaucescens* (**Figures 3E–H**) than in *M. paucispinus* (**Figures 4E–H**).

### Flow Cytometry

Flow cytometry analysis revealed four peaks of ploidy (2C, 4C, 8C, and 16C) in all topophysical zones of the stem in both species (**Table 1**). For *M. glaucescens*, the nuclear content varied from 2.97 (2C) to 23.56 pg DNA (~16C). In *M. paucispinus*, nuclear content was higher and ranged from 6.06 (2C) to 48.93 pg DNA (16C). No statistical difference in nuclear content was detected between cells from the apical, medial, and basal zones of the stem in both species (**Table 1**).

**Table 1:** Nuclear content (pg) from three topophysical zones of the stems of two *Melocactus* (Cactaceae) species.

	2C	4C	8C	16C
*Melocactus glaucescens*				
Apical	3.06a	6.06a	12.06a	23.56a
Medial	2.98a	5.85a	11.53a	22.46a
Basal	2.97a	5.81a	11.52a	22.40a
*Melocactus paucispinus*				
Apical	6.24a	12.44a	24.90a	48.93a
Medial	6.34a	12.66a	25.01a	48.89a
Basal	6.06a	12.15a	24.01a	47.97a

Means followed by the same lowercase letter in columns do not differ by Tukey’s test (P < 0.01).

As the above results were obtained using plants that had been cultured for ten years, an analogous set of experiments was performed comparing four- and ten-year cultures to determine if cells with mixed ploidy arose from extended cultivation or were a species-specific feature. Mixoploidy was observed in different regions of the stem and at both ages (**Table 2**). For *M. glaucescens*, no link was observed between stem regions and age, and ploidy levels did not differ statistically between ages (*P < 0.01*) (**Table 2**). However, an extra peak of 47 pg DNA (~32C) was observed in the cortex region. The roots were diploid and displayed only one peak of 2C (**Figure 2**, **Table 2**).

**Table 2:** Nuclear content (pg) of periphery, cortex, and stele regions of the stem, as well as the roots of two *Melocactus* (Cactaceae) species obtained from ten- and four-year old *in vitro* cultures.

	2C	4C	8C	16C		32C	
*Melocactus glaucescens*							
Ten years							
Periphery	3.17 aA	6.06 aA	13.06 aA	24.97 aA		–	
Cortex	3.06 aA	5.85 aA	12.09 aA	23.66 aA		46.79 A	
Stele	3.12 aA	5.81 aA	12.48 aA	24.22 aA		–	
Root	3.06 aA	–	–	–		–	
Four years							
Periphery	3.10 aA	6.01 aA	12.62 aA	24.24 aA		–	
Cortex	3.11 aA	5.95 aA	12.22 aA	24.00 aA		47.56 A	
Stele	3.26 aA	5.91 aA	12.65 aA	24.50 aA		–	
Root	3.04 aA	–	–	–		–	
*Melocactus paucispinus*							
Ten years							
Periphery	6.17 aA	12.06 aA	25.06 aA	47.97 aA		–	
Cortex	6.06 aA	12.85 aA	24.09 aA	46.66 aA		–	
Stele	6.12 aA	12.81 aA	24.48 aA	48.22 aA		–	
Root	6.06 aA	–	–	–		–	
Four years							
Periphery	6.15 aA	12.36 aA	25.10 aA	47.23 aA		–	
Cortex	6.09 aA	12.15 aA	24.02 aA	46.62 aA		–	
Stele	6.22 aA	12.10 aA	24.13 aA	48.13 aA		–	
Root	6.16 aA	–	–	–		–	

Means followed by the same lowercase letter in the stem regions and uppercase letter in age groups do not differ by Tukey’s test (P < 0.01).

A similar pattern of ploidy was observed in *M. paucispinus*, with no relationship between stem regions and age and no significant difference in ploidy between ages (*P < 0.01*) (**Table 2**). However, in contrast to *M. glaucescens*, the cortex region showed four peaks of ploidy in *M. paucispinus*. Likewise, the roots of *M. paucispinus* were also diploid (**Figure 2**, **Table 2**).

### X-Ray Micro-Computed Tomography

**Figure 5:**
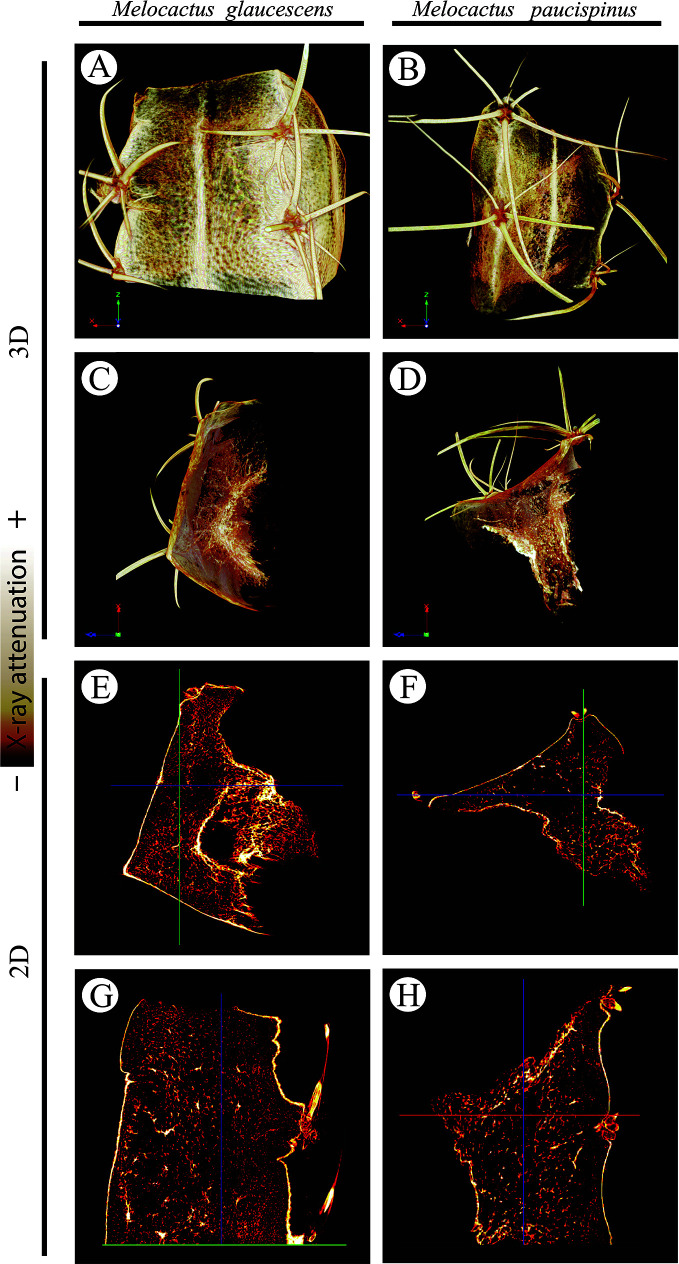
Three- (3D; **A–D**) two-dimensional (2D; **E–H**) reconstructions of *Melocactus glaucescens* (left column) and *Melocactus paucispinus* (right column) stems by X-ray micro-computed tomography, relying on the differential X-ray attenuation by the lumen and cell walls.

**Figure 6:**
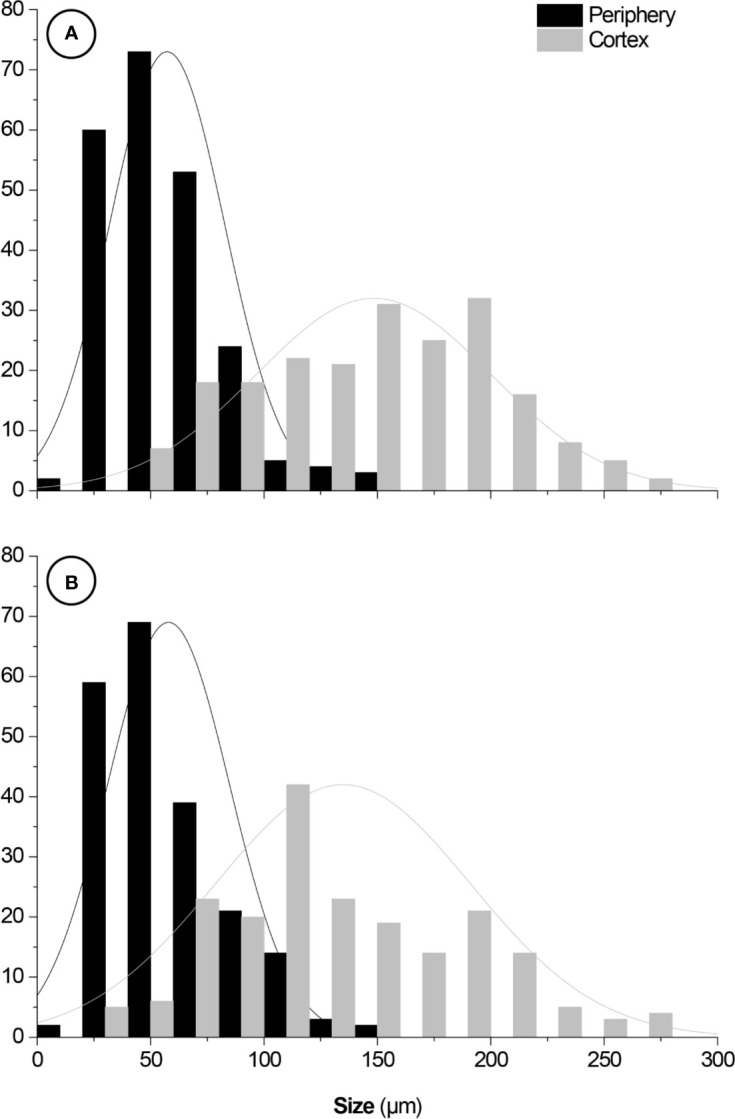
Size distributions of **(A)** *Melocactus glaucescens* and **(B)** *Melocactus paucispinus* cells from the periphery (57 ± 25 and 58 ± 27 µm, respectively) and the cortex (150 ± 54 and 135 ± 56 µm, respectively). Fittings: normal distributions. Y-axes: cell counts.

2D and 3D images indicated that cortical cells—darker (*i.e.*, lower X-ray attenuation) phases walled by brighter (*i.e.*, higher X-ray attenuation) constructs—were larger than the epidermal and hypodermal cells (**Figure 5**). 2D images of both species revealed that the cell diameter increased from the periphery (epidermis and hypodermis) towards the inner layers (aquifer and chlorophyll parenchyma) of the stem. The diameter of *M. glaucescens* and *M. paucispinus* cells differed (*p* < 0.05, *i.e.*, the null hypothesis was rejected and the populations are different at this level of significance) between the periphery (57 ± 25 and 58 ± 27 µm, respectively) and cortex of the stem (150 ± 54 and 135 ± 56 µm, respectively), which was accompanied by a smaller lumen in periphery cells (**Figure 6**). Accordingly, cortex cells were almost three times larger than the periphery cells in *M. glaucescens* and approximately two times in *M. paucispinus*. The cortex cells themselves did not differ in size (*p* > 0.05, *i.e.*, the populations are equal) among *M. glaucescens* and *M. paucispinus*, but the periphery cells in the former were significantly larger (*p* < 0.05) than in the latter.

## Discussion

The species investigated in this study belong to the same genus but differ in terms of 2C DNA content. *Melocactus glaucescens* contains approximately 3.0 pg DNA (2C), which corresponds to 2,934 Mbp (1 pg = 978 Mbp; Doležel et al., 2007). This value is very close to that of seven *Mammillaria* species (average of 3.13 pg) and *C. tenuissima* (2.87 pg) (Del Angel et al., 2006; Lema-Rumińska, 2011). However, compared to other cacti, this amount of nuclear DNA is one of the smallest reported and indicates a relatively small genome.

*Melocactus paucispinus* contains approximately 6.0 pg DNA (2C), corresponding to 5,868 Mbp, which is close to the content reported for six species of *Opuntia* (average of 6.51 pg) analyzed by Palomino et al. (2016). Karyotypes of *M. glaucescens* and *M. paucispinus* were previously documented by Assis et al. (2003) in a root apex cytogenetic-based study, which revealed the chromosome numbers of both species to be 2n = 44. In line with that report, we confirmed that the roots of *M. glaucescens* and *M. paucispinus* were diploid showing just two DNA peaks (2C and 4C) (**Figure 2**, **Table 2**).

As observed by Das et al. (1998a, 1998b) and Das and Mohanty (2008) the *Melocactus* genus is characterized by significant differences in DNA content among species, which can be conveniently detected by flow cytometry to identify species in natural populations and germplasm banks. Variations in ploidy between species are related to loss or addition of repeats in the genome, which may reflect micro- and macroscopic alterations during genome evolution (Das et al., 1998b). As a consequence, studies on ploidy have focused mostly on the systematics and evolution of this phenomenon (Bateman et al., 2018).

Only recently has the potential consequences of duplication events affecting specific cells or tissues been recognized, based on the use of homogenized tissues employed in flow cytometry analyses (Bateman et al., 2018). In this study, flow cytometry allowed the temporal, topological and cytological characterization of *M. glaucescens* and *M. paucispinus* stems and revealed mixed ploidy (mixoploidy) corresponding to 2C, 4C, 8C, 16C, and ~32C DNA content. Given that 16C and 32C DNA content was slightly lower than the exact values, it is possible that some DNA was lost during endocycles as had been suggested before for other cacti (Palomino et al., 2016) and several other non-cactus species as well (Ochatt, 2008; Ochatt and Abirached-Darmency, 2020).

Cells with lower DNA content (2C), such as those observed in the roots of these species, benefit from rapid genome replication and cell division (Scholes and Paige, 2015). This may translate into a considerable advantage for cacti as it allows for rapid recovery and proliferation of the roots during the sporadic and short rain seasons that bring life to an otherwise arid natural environment.

Endoreduplication-related events are often found in several plant cell types, especially in those undergoing differentiation and expansion, though a range of cell volumes are reached according to the response to environmental factors (Traas et al., 1998; Joubès and Chevalier, 2000). The onset of endoreduplication is a cytogenetic imprint of the transition from the embryogenic to the storage product accumulation phases during seed filling and within embryo development in species with small and large genome sizes (Ochatt and Abirached-Darmency, 2020). Smaller genomes favor also several rounds of endocycles (endoreduplication), which produce cells with higher DNA content and a different specialization (Bateman et al., 2018). In addition, endocycles occur predominantly in cells with large volumes (Breuer et al., 2014). Consistently, the higher ploidy of *M. glaucescens* and *M. paucispinus* cortex cells can be related to the presence of aquifer parenchyma, which is formed by large cells with elevated ploidy and capacity for storing large volumes of water (De Rocher et al., 1990). Indeed, endoreduplication has been associated with adaptation to saline and water stress conditions, extremely dry conditions and elevated temperatures (Lema-Rumińska, 2011; Elmaghrabi et al., 2013; Scholes and Paige, 2015; Elmaghrabi et al., 2017; Elmaghrabi et al., 2019).

For cells to become larger, they need to undergo discrete periods of S-phase and G-phase without cytokinesis (endocycle), which eventually results in cells with a single polyploid nucleus (Lee et al., 2009; Scholes and Paige, 2015). An increase in nuclear volume is often correlated with an increase in cell size as it ensures a functional surface-to-volume ratio. In this sense, endoreduplication offers an efficient strategy for plant growth (Kondorosi et al., 2000; Lee et al., 2009; Bhosale et al., 2019) and sometimes also favors repositioning of the nuclei within the cytoplasm under stress to better cope with it (Elmaghrabi et al., 2019).

In cacti, the growth caused by endoreduplication is related to the ability of parenchyma cells to expand and store water. Swelling and contraction occur naturally during the day. At night, CAM ensures that the stomata become open and the consequent transpiration causes a reduction in stem water potential and stem contraction (Wai et al., 2017). The nocturnal accumulation of malic acid by CAM and the daily synthesis of carbohydrates together increase the osmotic pressure in the vacuole of parenchyma cells, driving water into the cells and resulting in stem swelling during the day. The accumulation of inert osmolytes prevents any drastic metabolic effect so that cells can withstand stress (Scalisi et al., 2016; Wai et al., 2017; Gilman and Edwards, 2020).

Parenchyma cells, with their high capacity to expand when water is available, occupy 50–70% of the cortex volume in cacti (Barcikowski and Nobel, 1984). During periods of drought, secondary metabolites (*e.g.*, phenolic compounds and betalain) and polymerized sugars accumulate in the vacuoles of these cells to reduce water loss and prevent cellular damage caused by drought (Jain and Gould, 2015; Ventura-Aguilar et al., 2017). The volume of the stem decreases during the dry period. As reported by Lina and Eloisa (2018), *Melocactus curvispinus* plantlets could survive for eight months using water stored in their body, satisfying their hydric demands and maintaining carbon supply through photosynthesis.

The ability of parenchyma cells to expand and contract is related to their higher content of polysaccharides (*i.e.*, mucilage and pectin) compared to lignin. Despite not identifying typical mucilage cells, the cell walls showed some thickening, more evident in the cells of the epidermis and hypodermis, observed by ruthenium red and PAS staining for both *Melocactus* species (**Figures 3E–H** and **4E–H**). Here, acid floroglucin staining revealed the presence of lignin only in the spines and the helical thickening walls of the WBT (**Figures 3D** and **4D**). The slight presence of polysaccharides and lignin in the samples may be due to the *in vitro* cultivation. The polysaccharides set up a colloidal system to attract water and fill aquifer parenchyma cells, which thus become firm but flexible (Ventura-Aguilar et al., 2017). Together with the large volume of vacuoles caused by endoreduplicated cells, polysaccharides help create an extended system of water storage, which altogether can be considered the main elements responsible for conferring cellular support properties to succulent plants.

Four- and ten-year PGR-free *in vitro Melocactus* cultures exhibited a similar pattern of ploidy, akin to the peaks described by Lema-Rumińska (2011) in different tissues of *C. tenuissima*. Similar to *M. glaucescens*, long-term cultures of *Mammillaria san-angelis* did not show any change in ploidy with age of the culture and a maximum ploidy of ~32C was observed (Palomino et al., 1999). Consistency of the endopolyploidy pattern in cacti tissues regardless of the age of culture, further confirms that endoreduplication cycles in plants are genetically regulated to drive cell differentiation and offer a fine-tooled response during plant development (Cebolla et al., 1999; Nagymihály et al., 2017; Bateman et al., 2018; Bhosale et al., 2019). The endoreduplication program can differ among species of the same genus (Palomino et al., 2016). Indeed, the *Melocactus* species studied here exhibited different ploidy levels in the cortex region. *M. glaucescens* samples presented a peak of ~32C and cortex cells were almost three times the size of periphery cells; whereas *M. paucispinus* did not produce a peak of ~32C and cortex cells were only twice as large as periphery cells (**Figure 6** and **Table 2**).

Here, we report on the use of XRµCT to characterize the *Melocactus* stem from a morphoanatomical standpoint. The increase in cell volume observed in the samples is a consequence of endoreduplication. This was observed here by 2D and 3D XRµCT reconstructions, which demonstrate cellular lumen to increase in diameter from the periphery (epidermis and hypodermis) to the cortex (**Figure 6**). The larger cells were located in the cortical region, coinciding with the highest ploidy levels and water-storage capability of the aquifer parenchyma. Despite the increasing potential applications of this technique in plant developmental studies, the use in cacti is still scarce. Only recently, Kim et al. (2018) applied XRµCT to elucidate the hydraulic survival strategy of *Opuntia microdasys* and transport phenomena in the xylem structures of the root–stem junction, according to a hydrodynamic viewpoint.

Wide-band trachieds are a special type of tracheid with no perforations on their walls but instead possessing rigid secondary thickening bands (Mauseth, 1999). A region of WBTs was reported in several species of cacti, including in *Melocactus zehntneri* and *Melocactus bahiensis* (Mauseth et al., 1995; Mauseth and Plemons-Rodriguez, 1997; Mauseth, 1999; Stone-Palmquist and Mauseth, 2002; Arruda et al., 2004; Mauseth, 2004; Arruda et al., 2005; Mauseth, 2006; Melo-de-Pinna et al., 2006; Vázquez-Sánchez et al., 2007; Arruda and Melo-de-Pinna, 2010; Terrazas et al., 2016; Reyes-Rivera et al., 2018; Maceda et al., 2019). The paucity of support elements (*i.e.*, sclereids and fibers) indicates that the turgor pressure from the water stored in aquifer parenchyma, as well as epidermis, hypodermis, WBT present on vascular bundles in the cortex and pith provide structural support to the stem. Thus, the extreme specialization of the stem allows cacti to use the aquifer parenchyma not only for water storage but also for maintenance of the stem’s shape.

## Conclusion

This study provides new evidence on tissue organization and ploidy distribution in the stem of *Melocactus* species. As revealed by flow cytometry, anatomy, and XRµCT, the mixoploidy observed in both *Melocactus* species studied here is not related to age of the culture but is a characteristic intrinsic to the plant developmental program. Accordingly, endocycles promote cell differentiation, which in turn enables the accumulation of water, the most limiting natural resource in the environment where these plants live.

*Melocactus* has evolved a strategy to trigger quick growth when water becomes available again: the small genome in root cells enables rapid cell division cycles and endoreduplication of cortex cells, which are capable of a large expansion to optimize water storage.

## Data Availability Statement

The raw data supporting the conclusions of this article will be made available by the authors, without undue reservation.

## Author Contributions

GT-S, DB, CO, LV, AK, SR, and WO conceived and designed the study. GT-S performed the experiments. GT-S, WS, and AA carried out sample collection, preparation, and anatomical analyses. GT-S, EM, WS, and AK carried out sample collection, preparation, and flow cytometry analyses. GT-S, LC, and CO carried out collection, preparation, and XRµCT analyses. GT-S, DB, EF, and CO performed statistical analyses. GT-S, EM, DB, CO, AA, LV, AK, SR, CS, and WO wrote the manuscript.

## Funding

This work was supported by the Conselho Nacional de Desenvolvimento Científico e Tecnológico (CNPq, Brasília, DF, Brazil), Fundação de Amparo à Pesquisa do Estado de Minas Gerais (FAPEMIG, Belo Horizonte, Brazil), and Coordenação de Aperfeiçoamento de Pessoal de Nível Superior (CAPES, Brasília, DF, Brazil; Finance Code 001 and Internship Grant PDSE 88881.132727/2016-01 to GT-S), and Cornell University’s College of Agriculture and Life Sciences (GT-S, CS).

## Conflict of Interest

The authors declare that the research was conducted in the absence of any commercial or financial relationships that could be construed as a potential conflict of interest.

## Supplementary Material

The Supplementary Material for this article can be found online.

Supplementary Video 1 Three-dimensional X-ray micro-computed tomography reconstructions of a Melocactus glaucescens stem observed from different perspectives. The video is available at https://doi.org/10.6084/m9.figshare.12478271.v1

Supplementary Video 2 Three-dimensional X-ray micro-computed tomography reconstructions of a Melocactus paucispinus stem observed from different perspectives. The video is available at https://doi.org/10.6084/m9.figshare.12478271.v1
